# Supplementary material for: Kinesiology Students’ Perception Regarding Exercise Oncology: A Cross-Sectional Study
Source: Int J Environ Res Public Health. 2022 Jun 23;19(13):7724. doi: 10.3390/ijerph19137724 (PMC9265585; doi:10.3390/ijerph19137724)
Supplement: Supplementary file 1 [file ijerph-19-07724-s001.zip › ijerph-1746272-supplementary.pdf]

Section S1. Questionnaire to knowledge, perception, and expectations of exercise oncology

The following anonymous questionnaire investigates some aspects concerning the effect of physical activity in the oncology field. Read each statement and indicate your answers as accurately as possible. There are no right or wrong answers, just personal answers. Please, do not leave any questions unanswered.
Pursuant to EU Reg. 2016/679 or GDPR, "Code regarding the protection of personal data", we inform you that: (a) the answers provided will be used only in the research project context; (b) the data will be analysed for scientific purposes only; (c) the survey results will be published only in aggregate manner.

1. Age (years)

   ____

2. Sex (mark one answer only)
   - o Male
   - o Female

3. Weight (kilograms)

   ____

4. Height (meters)

   ____

5. Are you affected by, or have you been diagnosed with cancer? (mark one answer only)
   - o Yes
   - o No

6. Do you have or have you had a family member or a person close to you, who has or has had a diagnosis of cancer? (mark one answer only)
   - o Yes
   - o No

7. Which university are you enrolled in? (mark one answer only)
   - o University of Verona
   - o University of Rome “Foro Italico”
   - o University of Urbino
   - o University of Chieti-Pescara

8. Academic course (current attendance - mark one answer only)
   - o Bachelor's Degree program in Exercise and Sports Sciences (L-22)
   - o Master's Degree program in Health and Physical Activity (LM-67)
   - o Master's Degree program in Sports Sciences and Physical Performance (LM-68)
   - o Master's Degree program in Management of Sport (LM-47)

9. Current year of attendance (mark one answer only)
   - o 1
   - o 2
   - o 3
   - o 4
   - o 5

10. Have you ever been enrolled in advanced courses, training courses, and/or webinars on the "physical exercise and oncology" topic?
    - No
    - Yes (name it in the other section)

    Other section ____________________________________

11. What is the minimum amount of moderate-intensity aerobic exercise suggested for people who have or have been diagnosed with cancer, according to the American College of Sports Medicine? (mark one answer only)
    - At least 50 minutes per week
    - At least 90 minutes per week
    - At least 120 minutes per week
    - Aerobic activity is not recommended

12. What is the minimum amount of strength exercise suggested for people who have or have been diagnosed with cancer, according to the American College of Sports Medicine? (mark one answer only)
    - At least once per week
    - At least twice per week
    - At least 3 times per week
    - Strength activity is not recommended

13. Answer for each of the following statements / questions (mark only one answer per line)

	Absolutely yes	*Yes, rather than no*	*No, rather than yes*	*Absolutely no*
Do you think that physical activity is important to prevent breast, prostate and colorectum cancers?				
Are there teachings, in your degree program, that explain the prescription of physical activity and exercise as primary prevention of chronic non-communicable diseases?				
Are there teachings, in your degree program, that explain the physiopathology and treatments of cancer?				
Are there teachings, in your degree program, that explain the importance of psychological aspect in cancer?				

14. In your opinion, is exercise important and effective for psycho-physical health in patients with cancer?

	Absolutely yes	*Yes, rather than no*	*No, rather than yes*	*Absolutely no*

Before surgery (prehabilitation)				
During anticancer treatments (e.g., chemotherapy, radiotherapy, immunotherapy, hormone therapy)				
After anticancer treatments				

15. Are there teachings in your degree program that explain the prescription of physical activity and exercise for patients with cancer?

	Absolutely yes	*Yes, rather than no*	*No, rather than yes*	*Absolutely no*
Before surgery (prehabilitation)				
During anticancer treatments (e.g., chemotherapy, radiotherapy, immunotherapy, hormone therapy)				
After anticancer treatments				

16. Do you have sufficient knowledge and expertise for counselling physical activity to patients with cancer?

	Absolutely yes	*Yes, rather than no*	*No, rather than yes*	*Absolutely no*
Before surgery (prehabilitation)				
During anticancer treatments (e.g., chemotherapy, radiotherapy, immunotherapy, hormone therapy)				
After anticancer treatments				

17. Do you have sufficient knowledge and expertise to prescribe exercise in patients with cancer?

	Absolutely yes	*Yes, rather than no*	*No, rather than yes*	*Absolutely no*

Before surgery (prehabilitation)				
During anticancer treatments (e.g., chemotherapy, radiotherapy, immunotherapy, hormone therapy)				
After anticancer treatments				

18. Do you have sufficient knowledge and expertise to lead a group-based exercise training for patients with cancer?

	Absolutely yes	*Yes, rather than no*	*No, rather than yes*	*Absolutely no*
Before surgery (prehabilitation)				
During anticancer treatments (e.g., chemotherapy, radiotherapy, immunotherapy, hormone therapy)				
After anticancer treatments				

19. Do you have sufficient knowledge and expertise to lead an individual-based exercise training for patients with cancer?

	Absolutely yes	*Yes, rather than no*	*No, rather than yes*	*Absolutely no*
Before surgery (prehabilitation)				
During anticancer treatments (e.g., chemotherapy, radiotherapy, immunotherapy, hormone therapy)				
After anticancer treatments				

Table S1. Students' knowledge, perception, and confidence to "exercise oncology" theme according to academic degree.

	All (n=854)				*Academic course* *Bachelor's Degree in Sport and Exercise Science (n=497)*				*Master's Degree in Preventive and Adapted Exercise Science (n=285)*				*Other Master's Degree[1] (n=72)*			
	**Absolutely no**	**No, rather than yes**	**Yes, rather than no**	**Absolutely yes**	**Absolutely no**	**No, rather than yes**	**Yes, rather than no**	**Absolutely yes**	**Absolutely no**	**No, rather than yes**	**Yes, rather than no**	**Absolutely yes**	**Absolutely no**	**No, rather than yes**	**Yes, rather than no**	**Absolutely yes**
	%	%	%	%	%	%	%	%	%	%	%	%	%	%	%	%
Do you think that physical activity is important to prevent breast, prostate and colorectum cancers?	0	4	25	71	1	6	31	62	0	1	14	85	0	3	22	75
Are there teachings in your degree program that explain the prescription of physical activity and exercise as primary prevention of chronic non-communicable diseases?	13	25	26	36	1	32	27	25	4	10	26	60	22	36	21	21
Are there teachings in your degree program that explain the physiopathology and treatments of cancer?	22	38	25	15	25	40	25	10	12	33	29	26	43	39	10	8

Are there teachings in your degree program that explain the importance of psychological aspect in cancer?	32	38	19	11	39	36	17	8	21	40	23	16	33	44	14	8
Is exercise important and effective for psycho-physical health in patients with cancer?																
Before surgery	1	4	27	69	1	5	34	60	0	1	18	80	3	4	15	78
During anticancer treatments (chemotherapy, radiotherapy etc.)	2	13	32	52	2	16	36	45	1	9	26	63	1	8	33	57
After anticancer treatments	0	1	17	81	0	1	24	75	0	1	8	91	1	1	10	88
Are there teachings in your degree program that explain the prescription of physical activity and exercise for patients with cancer?																
Before surgery	24	36	23	16	28	39	21	12	12	31	31	27	50	35	13	3
During anticancer treatments (chemotherapy, radiotherapy etc.)	25	38	23	13	29	40	22	9	13	36	28	22	50	33	14	3
After anticancer treatments	23	33	25	19	26	36	23	15	12	28	31	29	46	38	14	3
Do you have sufficient knowledge and expertise for counselling physical activity to patients with cancer?																
Before surgery	29	40	23	7	39	39	16	6	11	40	37	12	35	43	18	4
During anticancer treatments (chemotherapy, radiotherapy etc.)	32	43	29	6	42	42	11	5	13	47	33	8	42	39	15	4
After anticancer treatments	28	38	26	9	36	39	17	7	11	35	41	13	36	36	22	6

Do you have sufficient knowledge and expertise to prescribe exercise in patients with cancer?																
Before surgery	31	40	21	7	40	40	15	5	13	41	35	11	42	40	15	3
During anticancer treatments (chemotherapy, radiotherapy etc.)	34	43	17	5	43	42	10	5	14	47	32	7	49	40	10	1
After anticancer treatments	30	39	23	8	38	40	15	7	12	38	38	12	43	36	18	3
Do you have sufficient knowledge and expertise to lead a group-based exercise training for patients with cancer?																
Before surgery	30	42	22	6	38	40	16	5	13	46	34	7	42	38	19	1
During anticancer treatments (chemotherapy, radiotherapy etc.)	32	46	17	5	41	44	10	5	14	52	30	5	46	38	15	1
After anticancer treatments	28	41	23	7	37	40	16	7	12	43	37	8	38	42	18	3
Do you have sufficient knowledge and expertise to lead an individual-based exercise training for patients with cancer?																
Before surgery	29	39	25	8	36	38	20	6	13	40	35	13	38	42	18	3
During anticancer treatments (chemotherapy, radiotherapy etc.)	32	43	19	6	40	42	12	6	15	46	31	8	46	40	13	1
After anticancer treatments	27	39	25	9	34	39	20	7	12	38	37	13	38	43	17	3

Table S2. Students' knowledge, perception, and confidence to "exercise oncology" theme according to academic degree and dividing 1st and 2nd year vs. 3rd year of bachelor's degree.

	Academic course															
	Bachelor's Degree in Sport and Exercise Science 1st and 2nd year (n=179)				***Bachelor'sDdegree in Sport and Exercise Science 3rd year (n=318)***				***Master's Degree in Preventive and Adapted Exercise Science (n=285)***				***Other Master's Degree[1] (n=72)***			
	**Absolutely no**	**No, rather than yes**	**Yes, rather than no**	**Absolutely yes**	**Absolutely no**	**No, rather than yes**	**Yes, rather than no**	**Absolutely yes**	**Absolutely no**	**No, rather than yes**	**Yes, rather than no**	**Absolutely yes**	**Absolutely no**	**No, rather than yes**	**Yes, rather than no**	**Absolutely yes**
	%	%	%	%	%	%	%	%	%	%	%	%	%	%	%	%
Do you think that physical activity is important to prevent breast, prostate, and colorectum cancers?	1	8	31	60	1	4	31	64	0	1	14	85	0	3	22	75
Are there teachings in your degree program that explain the prescription of physical activity and exercise as primary prevention of non-communicable diseases?	17	36	28	18	15	30	26	29	4	10	26	60	22	36	21	21
Are there teachings in your degree program that explain the physiopathology and treatments of cancer?	26	41	25	8	25	39	25	11	12	33	29	26	43	39	10	8
Are there teachings in your degree program that explain the importance of psychological aspect in cancer?	37	40	17	6	40	34	17	9	21	40	23	16	33	44	14	8
Is exercise important and effective for psycho-physical health in patients with cancer?																
Before surgery	1	6	36	58	1	5	32	62	0	1	18	80	3	4	15	78
During anticancer treatments (chemotherapy, radiotherapy etc.)	3	14	37	46	2	18	35	45	1	9	26	63	1	8	33	57
After anticancer treatments	1	1	18	80	0	1	27	72	0	1	8	91	1	1	10	88

Are there teachings, in your degree program, that explain the prescription of physical activity and exercise for patients with cancer?																
Before surgery	28	42	18	11	28	37	22	13	12	31	31	27	50	35	13	3
During anticancer treatments (chemotherapy, radiotherapy etc.)	30	40	22	7	28	40	21	11	13	36	28	22	50	33	14	3
After anticancer treatments	28	36	21	15	25	36	24	15	12	28	31	29	46	38	14	3
Do you have sufficient knowledge and expertise for counselling physical activity to patients with cancer?																
Before surgery	49	33	12	6	33	43	18	6	11	40	37	12	35	43	18	4
During anticancer treatments (chemotherapy, radiotherapy etc.)	50	34	10	6	37	46	12	5	13	47	33	8	42	39	15	4
After anticancer treatments	46	31	14	9	31	44	19	6	11	35	41	13	36	36	22	6
Do you have sufficient knowledge and expertise to prescribe exercise in patients with cancer?																
Before surgery	49	35	9	7	35	43	18	4	13	41	35	11	42	40	15	3
During anticancer treatments (chemotherapy, radiotherapy etc.)	53	32	8	7	38	47	11	4	14	47	32	7	49	40	10	1
After anticancer treatments	46	34	11	9	34	43	17	6	12	38	38	12	43	36	18	3
Do you have sufficient knowledge and expertise to lead a group-based exercise training for patients with cancer?																
Before surgery	49	33	13	5	32	44	18	5	13	46	34	7	42	38	19	1
During anticancer treatments (chemotherapy, radiotherapy etc.)	50	35	8	6	36	48	11	4	14	52	30	5	46	38	15	1
After anticancer treatments	45	34	13	8	32	43	18	7	12	43	37	8	38	42	18	3
Do you have sufficient knowledge and expertise to lead an individual-based exercise training for patients with cancer?																
Before surgery	47	32	15	6	31	41	23	6	13	40	35	13	38	42	18	3
During anticancer treatments (chemotherapy, radiotherapy etc.)	51	32	10	7	34	47	14	6	15	46	31	8	46	40	13	1
After anticancer treatments	44	35	13	8	29	41	24	6	12	38	37	13	38	43	17	3

Table S3. Comparison of students attending bachelor's degree and master's degree course with regard to knowledge, confidence of physical activity and exercise promotion in patients with cancer

Variables	Academic course	All (No.)	OR [1]	95% CI [2]	p-value [3]
Do you think that physical activity is important to prevent breast, prostate, and colorectum cancers?	Bachelor's Degree (reference)	497	Ref		
	Preventive and Adapted Exercise Science (Master's Degree)	285	6.68	2.03-21.99	0.002
	Other Master's Degree	72	2.49	0.58-10.60	0.217
Are there courses in your degree program that explain the prescription of physical activity and exercise as primary prevention of chronic non-communicable diseases?	Bachelor's Degree (reference)	497	Ref		
	Preventive and Adapted Exercise Science (Master's Degree)	285	5.29	3.79-8.07	<0.001
	Other Master's Degree	72	0.66	0.40-1.09	0.102
Are there courses in your degree program that explain the physiopathology and treatments of cancer?	Bachelor's Degree (reference)	497	Ref		
	Preventive and Adapted Exercise Science (Master's Degree)	285	2.28	1.69-3.07	<0.001
	Other Master's Degree	72	0.41	0.22-0.77	0.005
Are there courses in your degree program that explain the importance of psychological aspect in cancer?	Bachelor's degree (reference)	497	Ref		
	Preventive and Adapted Exercise Science (Master's Degree)	285	1.96	1.43-2.69	<0.001
	Other Master's Degree	72	0.83	0.46-1.50	0.530
Is exercise important and effective for psycho-physical health in patients with cancer?					
During anticancer treatments (chemotherapy, radiotherapy etc.)	Bachelor's degree (reference)	497	Ref		
	Preventive and Adapted Exercise Science (Master's Degree)	285	1.76	1.23-2.73	0.012
	Other Master's Degree	72	2.05	0.91-4.63	0.083
Are there courses in your degree program that explain the prescription of physical activity and exercise for patients with cancer?					

Before surgery	Bachelor's Degree (reference)	497	Ref		
	Preventive and Adapted Exercise Science (Master's Degree)	285	2.60	1.92-3.51	<0.001
	Other Master's Degree	72	0.36	0.18-0.70	0.003
During anticancer treatments (chemotherapy, radiotherapy etc.)	Bachelor's Degree (reference)	497	Ref		
	Preventive and Adapted Exercise Science (Master's Degree)	285	2.34	1.73-3.26	<0.001
	Other Master's Degree	72	0.43	0.22-0.82	0.010
After anticancer treatments	Bachelor's Degree (reference)	497	Ref		
	Preventive and Adapted Exercise Science (Master's Degree)	285	2.44	1.81-3.29	<0.001
	Other Master's Degree	72	0.33	0.17-0.62	0.001
Do you have sufficient knowledge and expertise for counselling physical activity to patients with cancer?					
Before surgery	Bachelor's Degree (reference)	497	Ref		
	Preventive and Adapted Exercise Science (Master's Degree)	285	3.38	2.46-4.64	<0.001
	Other Master's Degree	72	1.04	0.57-1.89	0.900
During anticancer treatments (chemotherapy, radiotherapy etc.)	Bachelor's Degree (reference)	497	Ref		
	Preventive and Adapted Exercise Science (Master's Degree)	285	3.28	2.35-4.59	<0.001
	Other Master's Degree	72	1.20	0.64-2.26	0.567
After anticancer treatments	Bachelor's Degree (reference)	497	Ref		
	Preventive and Adapted Exercise Science (Master's Degree)	285	3.61	2.65-4.93	<0.001
	Other Master's Degree	72	1.18	0.68-2.06	0.554
Do you have sufficient knowledge and expertise to prescribe exercise in patients with cancer?					

Before surgery	Bachelor's Degree (reference)	497	Ref		
	Preventive and Adapted Exercise Science (Master's Degree)	285	3.23	2.34-4.46	<0.001
	Other Master's Degree	72	0.88	0.47-1.68	0.704
During anticancer treatments (chemotherapy, radiotherapy etc.)	Bachelor's Degree (reference)	497	Ref		
	Preventive and Adapted Exercise Science (Master's Degree)	285	3.30	2.34-4.66	<0.001
	Other Master's Degree	72	0.69	0.32-1.50	0.346
After anticancer treatments	Bachelor's Degree (reference)	497	Ref		
	Preventive and Adapted Exercise Science (Master's Degree)	285	3.57	2.60-4.89	<0.001
	Other Master's Degree	72	0.93	0.51-1.71	0.815
Do you have sufficient knowledge and expertise to lead a group-based exercise training for patients with cancer?					
Before surgery	Bachelor's Degree (reference)	497	Ref		
	Preventive and Adapted Exercise Science (Master's Degree)	285	2.45	1.78-3.38	<0.001
	Other Master's Degree	72	0.96	0.52-1.76	0.885
During anticancer treatments (chemotherapy, radiotherapy etc.)	Bachelor's Degree (reference)	497	Ref		
	Preventive and Adapted Exercise Science (Master's Degree)	285	2.83	2.00-4.01	<0.001
	Other Master's Degree	72	1.11	0.57-2.16	0.770
After anticancer treatments	Bachelor's Degree (reference)	497	Ref		
	Preventive and Adapted Exercise Science (Master's Degree)	285	2.55	1.87-3.47	<0.001
	Other Master's Degree	72	0.76	0.42-1.96	0.382
Do you have sufficient knowledge and expertise to lead an individual-based exercise training for patients with cancer?					

Before surgery	Bachelor's Degree (reference)	497	Ref		
	Preventive and Adapted Exercise Science (Master's Degree)	285	2.55	1.87-3.47	<0.001
	Other Master's Degree	72	0.76	0.42-1.96	0.382
During anticancer treatments (chemotherapy, radiotherapy etc.)	Bachelor's Degree (reference)	497	Ref		
	Preventive and Adapted Exercise Science (Master's Degree)	285	2.75	1.97-3.82	<0.001
	Other Master's Degree	72	0.72	0.35-1.45	0.355
After anticancer treatments	Bachelor's Degree (reference)	497	Ref		
	Preventive and Adapted Exercise Science (Master's Degree)	285	2.71	2.00-3.69	<0.001
	Other Master's Degree	72	0.66	0.35-1.22	0.183

[1] OR (odds ratios), [2] CI (confidence intervals), and [3] *p*-values from multivariable logistic regression model. Data about the importance and the effectiveness of exercise on psycho-health of patients with cancer resulted non significative in the univariate analysis.

Table S4. Comparison of students attending bachelor's degree (1st and 2nd vs 3rd year) and master's degree courses with regard to knowledge, confidence of physical activity, and exercise promotion in patients with cancer

Variables	Academic course	All (No.)	OR [1]	95% CI [2]	*p*-value [3]
Do you think that physical activity is important to prevent breast, prostate, and colorectum cancers?	Bachelor's Degree 3rd year (reference)	179	Ref		
	Bachelor's Degree 1st and 2nd year	318	0.51	0.25-1.03	0.06
	Preventive and Adapted Exercise Science (Master's Degree)	285	4.98	1.44-17.27	0.01
	Other Master's Degree	72	1.85	0.42-8.25	0.42
Are there courses in your degree program that explain the prescription of physical activity and exercise as primary prevention of chronic non-communicable diseases?	Bachelor's Degree 3rd year (reference)	179	Ref		
	Bachelor's Degree 1st and 2nd year	318	0.71	0.50-1.02	0.06
	Preventive and Adapted Exercise Science (Master's Degree)	285	5.01	3.53-7.47	<0.001
	Other Master's Degree	72	0.58	0.35-0.98	0.042
Are there courses in your degree program that explain the physiopathology and treatments of cancer?	Bachelor's Degree 3rd year (reference)	179	Ref		
	Bachelor's Degree 1st and 2nd year	318	0.87	0.59-1.28	0.47
	Preventive and Adapted Exercise Science (Master's Degree)	285	2.17	1.56-3.00	<0.001
	Other Master's Degree	72	0.40	0.21-0.74	0.004
Are there courses in your degree program that explain the importance of psychological aspect in cancer?	Bachelor's Degree 3rd year (reference)	179	Ref		
	Bachelor's Degree 1st and 2nd year	318	0.87	0.56-1.33	0.52
	Preventive and Adapted Exercise Science (Master's Degree)	285	1.83	1.30-2.60	<0.001
	Other Master's Degree	72	0.81	0.44-1.49	0.50
Is exercise important and effective for psycho-physical health in patients with cancer?					
During anticancer treatments (chemotherapy, radiotherapy etc.)	Bachelor's Degree 3rd year (reference)	179	Ref		
	Bachelor's Degree 1st and 2nd year	318	0.84	0.39-1.78	0.64
	Preventive and Adapted Exercise Science (Master's Degree)	285	4.22	1.41-12.61	0.01
	Other Master's Degree	72	0.80	0.29-2.24	0.68
Are there courses in your degree program that explain the prescription of physical activity and exercise for patients with cancer?					

Before surgery	Bachelor's Degree 3rd year (reference)	179	Ref		
	Bachelor's Degree 1st and 2nd year	318	1.28	0.76-1.98	0.40
	Preventive and Adapted Exercise Science (Master's Degree)	285	2.02	1.27-3.22	0.003
	Other Master's Degree	72	2.29	1.01-5.25	0.05
During anticancer treatments (chemotherapy, radiotherapy etc.)	Bachelor's Degree 3rd year (reference)	179	Ref		
	Bachelor's Degree 1st and 2nd year	318	1.13	0.20-6.22	0.89
	Preventive and Adapted Exercise Science (Master's Degree)	285	0.71	0.19-2.68	0.61
	Other Master's Degree	72	0.44	0.08-2.48	0.35
After anticancer treatments	Bachelor's Degree (reference)	179	Ref		
	Bachelor's Degree 1st and 2nd year	318	0.76	0.51-1.13	0.18
	Preventive and Adapted Exercise Science (Master's Degree)	285	2.42	1.75-3.37	<0.001
	Other Master's Degree	72	0.38	0.17-0.65	0.001
Do you have sufficient knowledge and expertise for counselling physical activity to patients with cancer?					
Before surgery	Bachelor's Degree 3rd year (reference)	179	Ref		
	Bachelor's Degree 1st and 2nd year	318	0.89	0.60-1.33	0.57
	Preventive and Adapted Exercise Science (Master's Degree)	285	2.19	1.58-3.05	<0.001
	Other Master's Degree	72	0.42	0.22-0.82	0.01
During anticancer treatments (chemotherapy, radiotherapy etc.)	Bachelor's Degree 3rd year (reference)	179	Ref		
	Bachelor's Degree 1st and 2nd year	318	0.89	0.61-1.30	0.56
	Preventive and Adapted Exercise Science (Master's Degree)	285	2.35	1.69-3.26	<0.001
	Other Master's Degree	72	0.31	0.16-0.61	<0.001
After anticancer treatments	Bachelor's Degree 3rd year (reference)	179	Ref		
	Bachelor's Degree 1st and 2nd year	318	0.71	0.44-1.12	0.13
	Preventive and Adapted Exercise Science (master's degree)	285	3.09	2.18-4.37	0.001
	Other Master's Degree	72	0.93	0.50-1.71	0.81
Do you have sufficient knowledge and expertise to prescribe exercise in patients with cancer?					

Before surgery	Bachelor's Degree 3rd year (reference)	179	Ref		
	Bachelor's Degree 1st and 2nd year	318	0.89	0.54-1.46	0.64
	Preventive and Adapted Exercise Science (Master's Degree)	285	3.24	2.22-4.71	<0.001
	Other Master's Degree	72	1.15	0.60-2.22	0.67
During anticancer treatments (chemotherapy, radiotherapy etc.)	Bachelor's Degree 3rd year (reference)	179	Ref		
	Bachelor's Degree 1st and 2nd year	318	0.87	0.57-1.34	0.52
	Preventive and Adapted Exercise Science (Master's Degree)	285	3.44	2.44-4.85	<0.001
	Other Master's Degree	72	1.13	0.63-1.99	0.69
After anticancer treatments	Bachelor's Degree 3rd year (reference)	179	Ref		
	Bachelor's Degree 1st and 2nd year	318	0.69	0.43-1.11	0.12
	Preventive and Adapted Exercise Science (Master's Degree)	285	2.93	2.06-4.17	<0.001
	Other Master's Degree	72	0.78	0.41-1.51	0.46
Do you have sufficient knowledge and expertise to lead a group-based exercise training for patients with cancer?					
Before surgery	Bachelor's Degree 3rd year (reference)	179	Ref		
	Bachelor's Degree 1st and 2nd year	318	0.98	0.59-1.63	0.92
	Preventive and Adapted Exercise Science (Master's Degree)	285	3.40	2.31-5.01	<0.001
	Other Master's Degree	72	0.67	0.31-1.52	0.35
During anticancer treatments (chemotherapy, radiotherapy etc.)	Bachelor's Degree 3rd year (reference)	179	Ref		
	Bachelor's Degree 1st and 2nd year	318	0.88	0.56-1.38	0.56
	Preventive and Adapted Exercise Science (Master's Degree)	285	3.46	2.43-4.91	<0.001
	Other Master's Degree	72	0.92	0.49-1.71	0.78
After anticancer treatments	Bachelor's Degree 3rd year (reference)	179	Ref		
	Bachelor's Degree 1st and 2nd year	318	0.75	0.47-1.18	0.21
	Preventive and Adapted Exercise Science (Master's Degree)	285	2.30	1.61-3.26	<0.001
	Other Master's Degree	72	0.87	0.46-1.62	0.66

Do you have sufficient knowledge and expertise to lead an individual-based exercise training for patients with cancer?					
Before surgery	Bachelor's Degree 3rd year (reference)	179	Ref		
	Bachelor's Degree 1st and 2nd year	318	0.66	0.43-1.02	0.06
	Preventive and Adapted Exercise Science (Master's Degree)	285	2.28	1.63-3.19	<0.001
	Other Master's Degree	72	0.67	0.36-1.23	0.20
During anticancer treatments (chemotherapy, radiotherapy etc.)	Bachelor's Degree 3rd year (reference)	179	Ref		
	Bachelor's Degree 1st and 2nd year	318	0.85	0.52-1.37	0.50
	Preventive and Adapted Exercise Science (Master's Degree)	285	2.69	1.86-3.88	<0.001
	Other Master's Degree	72	0.68	0.33-1.40	0.30
After anticancer treatments	Bachelor's Degree 3rd year (reference)	179	Ref		
	Bachelor's Degree 1st and 2nd year	318	0.66	0.43-1.02	0.06
	Preventive and Adapted Exercise Science (Master's Degree)	285	2.43	1.74-3.40	<0.001
	Other Master's Degree	72	0.58	0.31-1.08	0.09

[1] OR (odds ratios), [2] CI (confidence intervals), and [3] *p*-values from multivariable logistic regression model, [4] knowledge and confidence defined as absolutely yes/ yes, rather than no vs. no, rather than yes/ absolutely no
